# Grade group system and plasma androgen receptor status in the first line treatment for metastatic castration resistant prostate cancer

**DOI:** 10.1038/s41598-022-10751-6

**Published:** 2022-05-05

**Authors:** M. C. Cursano, V. Conteduca, E. Scarpi, G. Gurioli, C. Casadei, S. Gargiulo, A. Altavilla, C. Lolli, B. Vincenzi, G. Tonini, D. Santini, U. De Giorgi

**Affiliations:** 1 Department of Medical Oncology, Campus Bio-Medico University, via Alvaro del Portillo, 200, 00128 Rome, Italy; 2 Department of Medical Oncology, Istituto Scientifico Romagnolo per lo Studio e la Cura dei Tumori (IRST) IRCCS, via Maroncelli, 40, 47014 Meldola, Italy; 3 Unit of Biostatistics and Clinical Trials, Istituto Scientifico Romagnolo per lo Studio e la Cura dei Tumori (IRST) IRCCS, Meldola, Italy; 4 Biosciences Laboratory, Istituto Scientifico Romagnolo per lo Studio e la Cura dei Tumori (IRST) IRCCS, Meldola, Italy

**Keywords:** Cancer, Biomarkers, Oncology, Urology

## Abstract

In localized prostate cancer (PCa), Grade Group (GG) and Gleason Score (GS) have a well-established prognostic role. In metastatic castration resistant prostate cancer (mCRPC), the prognostic role of GS and GG is less defined. In first-line treatment of mCRPC, androgen receptor (AR)-directed drugs (abiraterone acetate, enzalutamide) and docetaxel represent the referring options. There is no evidence that the GS/GG systems can add information to guide the choice between AR-directed drugs and docetaxel in the first-line setting of mCRPC. Nowadays there are no validated biomarkers, which define patients who may benefit or not from hormonal treatments or chemotherapy. Androgen receptor (AR) copy number variations (CNV) are predictive factors of poor response to abiraterone and enzalutamide. There are no available data about the association between AR CNV and GG. In this retrospective study, we analysed the association of the highest GG score with AR CNV and their impact on the clinical outcome of AR-directed drugs and docetaxel as first-line therapy for mCRPC patients. Patients benefit from docetaxel, abiraterone or enzalutamide regardless the GG. However, the presence of GG5 and AR CNV gain identifies a subgroup of patients with poor prognosis, which could benefit from front-line docetaxel instead of AR-directed drugs.

## Introduction

Since 1996, the Gleason score system (GS) provides a risk stratification in men with localized prostate cancer (PCa) basing on histologic architectural patterns^1^. In localised PCa, GS system predicts biochemical relapse, metastatic disease-free survival and overall survival (OS)^2^. In 2005 and more recently in 2014, the International Society of Urological Pathology (ISUP) proposed the Grade Group system (GG) to be used in parallel with GS. GG identifies five distinct risk categories: GG 1 corresponding to GS ≤ 6, GG 2 to GS 3 + 4 = 7, GG 3 to GS 4 + 3 = 7, GG 4 to GS 4 + 4 = 8, and GG 5 to GS 9/10^3,4^. Conventionally, GS ≥ 8 as well as GG 4–5 identified men with high-risk PCa^5^. After the definition of the GG system, more relevance has been given to the difference between GS 8 (GG 4) and GS 9–10 (GG5) PCa. Recent studies demonstrated that GS 9–10/GG 5 hormone sensitive PCa may benefit less from androgen deprivation therapy (ADT) and should be treated with lifelong ADT, on the contrary, GS 8 or GG4 PCa may benefit from short term ADT or long term ADT^6,7^. Contrary to what occurs in localized PCa, the prognostic role of GS grading systems and GG in metastatic castration resistant prostate cancer (mCRPC) is less defined. In first-line treatment of mCRPC, androgen receptor (AR)-directed drugs (abiraterone acetate, enzalutamide) and docetaxel represent the referring options^8–13^. A recent study investigated the role of GS at diagnosis as a predictor of response to abiraterone in mCRPC patients enrolled in clinical trials of abiraterone in the pre and post-docetaxel setting (COU-AA-302 and COU-AA-301, respectively). Abiraterone significantly improved outcomes in mCRPC independently from GS ≥ 8 vs GS < 8^14^. There are no further data to support the role of GS and GG as prognostic or predictive factors of response to treatment in patients with mCRPC; consequently, there is no clear evidence that the GS/GG systems can add information to guide the choice between AR-directed drugs and docetaxel in the first line setting of mCRPC. Nowadays there are no validated further biomarkers, which define patients who may benefit or not from hormonal treatments or chemotherapy. Several studies evaluated the role androgen receptor (AR) aberrations as prognostic and predictive factors of response to treatments. AR aberrations are later events, which occur in the castration resistance phase. In fact, AR aberrations are present in more than 60% of biopsies on metastatic sites of mCRPC patients^15^. On the contrary, no alterations of the AR gene were found in the neoadjuvant setting^16–18^. Among the AR aberrations, AR copy number variations (CNV) correlate with clinical outcome in mCRPC patients treated with AR-directed therapies (abiraterone and enzalutamide), whereas no association was observed in patients treated with taxanes^19,20^. There are no available data about the association between AR CNV and GS/GG at diagnosis. In this study, we analysed the association of the highest GG score with AR CNV and their impact on the clinical outcome of AR-directed drugs and docetaxel as first-line therapy in mCRPC patients.

## Results

### Patient characteristics

Among the 242 patients with mCRPC eligible for this study, 165 and 77 patients had GG 2–4 and GG 5 at diagnosis, respectively. Among patients with GG 2–4, 74 (44.8%) patients received a first-line treatment with docetaxel, 91 (55.2%) patients received abiraterone (n = 44) or enzalutamide (n = 47). Among patients with GG5, docetaxel was performed by 37 (48%) patients, the remaining 40 (52%) patients underwent abiraterone (n = 18) or enzalutamide (n = 22) as first-line treatment for mCRPC. Baseline characteristics of patients with GS 7–8 (GG2–4) and GS 9–10 (GG5) disease are described in Table 1. There were not significantly differences in age, ECOG PS, primary treatment, baseline value of LDH, chromogranin A and alkaline phosphatase (ALP) in patients with GG 2–4 and GG 5 treated with abiraterone/enzalutamide or docetaxel. Patients with GG5 presented greater tumor burden (in terms of visceral, lymph nodes and bone metastases) compared to those patients with GG 2–4 (Table 1). Patients with GG 5 presented shorter time from initial diagnosis to the beginning of treatment for mCRPC than patients with GG 2–4.

**Table 1:** Baseline patients characteristics.

First line mCRPC	Docetaxel		*p* value	Abiraterone or enzalutamide		*p* value
	Grade group 2–4	Grade group 5		Grade group 2–4	Grade group 5	
No. patients	74	37		91	40	
Age, median (IQR), years	72 (67–78)	68 (62–74)	0.06	76 (70–81)	74 (66–79)	0.09
**ECOG PS, n (%)**						
0	47 (74.6)	28 (84.9)		76 (84.5)	27 (75.0)	
1	14 (22.2)	4 (12.1)		10 (11.1)	6 (16.7)	
2	2 (3.2)	1 (3.0)	0.33	4 (4.4)	3 (8.3)	0.21
**Primary treatment**						
Radiation	15 (30.0)	4 (23.5)		20 (35.1)	3 (14.3)	
RP	35 (70.0)	13 (76.5)	0.61	37 (64.9)	18 (85.7)	0.07
**Extent of disease, n (%)**						
Bone only	38 (51.3)	10 (27.0)		30 (34.1)	9 (22.5)	
Lymphnode only	11 (14.9)	6 (16.2)		30 (34.1)	5 (12.5)	
Lymphnode + bone	15 (20.3)	16 (43.3)		24 (27.3)	23 (57.5)	
Visceral	10 (13.5)	5 (13.5)	0.03	4 (4.5)	3 (7.5)	0.00
Baseline PSA, median (IQR), ng/ml	32.20 (13.15–95.29)	17.85 (8.67–44.80)	0.25	7.72 (3.22–29.94)	23.00 (5.97–49.28)	0.01
Baseline LDH, median (IQR), IU/l	171 (158–208)	195 (155–230)	0.26	183 (167–204)	189 (168–226)	0.47
Baseline chromogranin, median (IQR), µg/l	165 (65–280)	98 (58–175)	0.82	66 (37–212)	75 (31–144)	0.75
Baseline ALP median (IQR), IU/l	95 (78–150)	104 (68–161)	0.53	95 (68–110)	97 (60–137)	0.78
Time from initial diagnosis to first dose (IQR), months	62.0 (26.5–111.8)	28.1 (19.5–53.7)	0.003	78.4 (37.0–151.4)	31.2 (16.7–79.3)	0.003

*IQR* Interquartile range, *ECOG PS* Eastern Cooperative Oncology Group Performance Status, *PSA* prostate specific antigen, *LDH* lactate dehydrogenase, *ALP* alkaline phosphatase, *n* number.

### Clinical outcomes after abiraterone/enzalutamide or docetaxel according to grade group system

The median follow up at the time of analysis was 47 months (range 2–109). In patients receiving docetaxel as first-line treatment, the univariate analysis showed that GG did not significantly influence OS (p = 0.09); median OS was 37.6 months (95% CI 30.5–48.2) in patients with GG 2–4 and 29.8 months (95% CI 22.2–46.7) in GG 5. Similarly, median PFS was not significantly different (p = 0.55) for patients with GG 2–4 (10.6 months 95% CI 8.9–11.5) compared to GG 5 (9.0 months 95% CI 7.3–10.9) disease. Chemotherapy-naive mCRPC treated with abiraterone or enzalutamide presented no significantly difference for median OS (p = 0.69) and PFS (p = 0.22) depending on GG 2–4 and GG 5 (Table 2). GG did not significantly affect PSA response rate in chemotherapy-naïve mCRPC patients treated with abiraterone or enzalutamide (p = 0.57) as well as in docetaxel-treated patients (p = 0.34) (Table 2).

**Table 2:** Prostate-specific antigen (PSA) response rate and progression-free survival (PFS) and overall survival (OS) according to Grade Group 2–4 versus Grade Group 5 and treatment.

First line mCRPC	Docetaxel		*p* value	Abiraterone or enzalutamide		*p* value
	Grade group 2–4	Grade group 5		Grade group 2–4	Grade group 5	
PSA response rate (%)	44 (72.1)	20 (62.5)	0.34	62 (72.1)	20 (66.7)	0.57
Median PFS, months (95% CI)	10.6 (8.9–11.5)	9.0 (7.3–10.9)	0.55	16.6 (11.9–18.7)	10.8 (7.8–17.6)	0.22
Median OS, months (95% CI)	37.6 (30.5–48.2)	29.8 (22.2–46.7)	0.09	29.8 (27.1–50.5)	44.2 (20.7–52.8)	0.69

*PSA* Prostate-specific antigen, *PFS* Progression-free survival, *OS* Overall survival, *mCRPC* metastatic castration-resistant prostate cancer, *nr* not reached.

### Clinical outcomes patients according to AR CNV status and grade group

Blood samples for AR CNV detection were available from 164 patients (116 samples in GG 2–4 and 48 samples in GG 5 mCRPC) at baseline of the first-line treatment with docetaxel or AR-directed agents (enzalutamide or abiraterone). In docetaxel treated patients, AR CNV gain was detected in 15 and 8 blood samples of patients with GG 2–4 and GG 5 disease, respectively (Table 4). In these patients, AR CNV normal was observed in 32 and 15 blood samples of patients with GG 2–4 and GG 5, respectively. In patients treated with abiraterone or enzalutamide, AR CNV normal was identified in 61 patients with GG 2–4 and 18 patients with GG 5. Eight patients with GG 2–4 and 7 patients with GG 5 had AR CNV gain at baseline of abiraterone or enzalutamide (Table 3). Overall, 20% of patients with GG 2–4 resulted AR CNV gain at baseline compared with 31% of patients with GG 5; nevertheless, this difference was not significant.

**Table 3:** Grade group at diagnosis by treatment groups and AR copy number status.

First line mCRPC	*n* (%)	AR copy number		
		Normal	Gain	Not available
**Docetaxel, *N***				
Grade group 2–4	74 (66.7)	32	15	27
Grade group 5	37 (33.3)	15	8	14
**Abiraterone, *N***				
Grade group 2–4	44 (71.0)	26	4	14
Grade group 5	18 (29.0)	7	2	9
**Enzalutamide, *N***				
Grade group 2–4	47 (68.1)	35	4	8
Grade group 5	22 (31.9)	11	5	6

*mCRPC* metastatic castration-resistant prostate cancer, *AR* androgen receptor, *n* number.

The univariate analysis of median PFS and median OS according to GG and AR CNV status in patients treated with docetaxel and in those patients treated with abiraterone or enzalutamide is shown in Table 4. In both AR CNV normal and gain patients, no significant difference in median PFS was shown in both patients treated with docetaxel (normal: HR 1.41 (0.75–2.65), p = 0.28; gain: HR 0.63 (0.24–1.66), p = 0.35) and abiraterone or enzalutamide (normal: HR 0.93 (0.44–1.93), p = 0.84; gain: HR 1.21 (0.36–4.04), p = 0.75), depending on the GG 2–4 or GG 5. Similarly, in docetaxel-treated patients, no difference in OS was found in patients with GG 2–4 and GG 5 mCRPC depending on normal (HR 1.71 (0.87–3.36), p = 0.11) or gain (HR 0.60 (0.21–1.68), p = 0.32) status of AR CNV. Chemotherapy-naïve patients treated with abiraterone or enzalutamide with AR CNV gain and GG5 experienced a significantly worsening in median OS (20.2 months in GG 2–4 vs 7.8 months in GG 5, p 0.04). No difference in median OS depending on GG 2–4 or GG 5 was observed for abiraterone or enzalutamide- treated patients with AR CNV normal at baseline (HR 1.03 (0.44–2.43), p = 0.94).

**Table 4:** Univariate analysis of PFS and OS according to treatment, AR status and Grade Group 2–4 versus 5.

	PFS						OS				
	n. pts	n. events	Median PFS (months) (95% CI)	p	HR (95% CI)	p	n. events	Median OS (months) (95% CI)	p	HR (95% CI)	p
ABI/ENZA	94	58	16.2 (10.1–18.9)	–	–	–	40	27.4 (21.0–43.8)	–	–	–
**AR Normal**											
Grade group 2–4	61	36	17.1 (11.9–22.3)		1.00		22	36.7 (24.5–nr)		1.00	
Grade group 5	18	10	16.6 (8.6–51.5)	0.83	0.93 (0.44–1.93)	0.84	7	44.2 (18.3–52.8)	0.94	1.03 (0.44–2.43)	0.94
**AR gain**											
Grade group 2–4	8	7	6.6 (1.6–35.8)		1.00		5	20.2 (4.0–35.8)		1.00	
Grade group 5	7	5	6.6 (2.8–13.7)	0.75	1.21 (0.36–4.04)	0.75	5	7.8 (6.0–13.7)	0.02	4.92 (0.92–26.27)	0.04
Docetaxel	70	70	9.9 (8.9–11.0)	–	–	–	59	35.4 (28.7–44.5)	–	–	–
**AR normal**											
Grade group 2–4	32	32	10.6 (8.4–11.4)		1.00		25	40.4 (28.7–57.8)		1.00	
Grade group 5	15	15	8.5 (5.8–10.8)	0.28	1.41 (0.75–2.65)	0.28	14	29.8 (14.9–49.2)	0.11	1.71 (0.87–3.36)	0.11
**AR gain**											
Grade group 2–4	15	15	9.8 (3.5–12.7)		1.00		13	31.9 (20.0–44.5)		1.00	
Grade group 5	8	8	10.1 (6.5–30.3)	0.34	0.63 (0.24–1.66)	0.35	7	33.0 (17.3–107.0)	0.32	0.60 (0.21–1.68)	0.32

*AR* androgen receptor, *ABI* abiraterone, *ENZA* enzalutamide, *PFS* progression free survival, *OS* overal survival, *nr* not reached, *ne* not estimable, *HR* hazard ratio, *n* number, *nr* not reached, *ne* not estimable.

## Discussion

The GS system and the latest GG system provide a risk assessment in men with localized prostate cancer by predicting biochemical recurrence, development of metastasis and overall survival^2,3^. In localized hormone-sensitive PCa, the presence of GG 4 and GG 5 identifies worse prognosis patients. Recent studies investigated whether hormone-sensitive PCa with GG 4 could present a different clinical outcome with ADT than GG 5. A retrospective analysis of patients with localized hormone-sensitive PCa stated that GG 5 disease derives less benefit from ADT^6^. Furthermore, Kishan et al., in a recent meta-analysis, suggested that GG 4 disease could benefit from short term and long term ADT, while GG 5 benefit from lifelong ADT^7^.

Contrary to what occurs in localized hormone-sensitive PCa, the prognostic role of GS and GG grading systems in mCRPC is less defined. A recent study investigated the role of GS at diagnosis as a predictor of response to abiraterone in patients with mCRPC enrolled in clinical trials of abiraterone in the pre and post-docetaxel setting (COU-AA-302 and COU-AA-301, respectively). The results of this retrospective analysis showed that patients with mCRPC benefit from abiraterone regardless of GS < or ≥ 8^14^.

In our retrospective study, we identified patients with mCRPC and GG at diagnosis 2–5 who underwent first-line treatment, stratifying them by GG (2–4 versus 5) and by type of treatment (docetaxel versus abiraterone or enzalutamide).

The results of this study confirm that stratifying patients according to GG 2–4 and GG 5 no differences were found in median OS and median PFS both in the subgroup of patients treated with docetaxel and those patients treated with AR-directed drugs (abiraterone or enzalutamide). According to the results of our analysis, the GG grading system, therefore, is not a predictive factor of response to the mCRPC first-line treatment with docetaxel or abiraterone/enzalutamide.

Grade group 5 PCa would require intensified treatment due to aggressive behaviour and early tendency to develop mechanisms of resistance to ADT. The biological explanation of the potential insensitivity of GG 5 to ADT remains unknown. There are no clear histo-morphological reasons why GG 5 diseases should be resistant to ADT. However, the poorly differentiated phenotype is likely to be associated with activation of cell survival signals, which are independent from AR pathway. Frequently, these poorly differentiated histotypes are associated to low PSA values and characteristics of neuroendocrine differentiation^7^. In fact, PCa with pathologically confirmed neuroendocrine differentiation is characterised by low PSA levels, visceral metastases and loss of RB1 and TP53 genes (suggesting less AR-driven disease)^21^. Similarly, GG 5 PCa are characterized by high genomic instability and alterations in the main signaling pathways (TP53, PTEN and RB) involved in the resistance to hormonal therapies. In particular, TP53 alterations are frequent in GG5 prostate tumors and are associated to rapid progression and evolution towards the castration resistance phase^22^. Hormone-resistance mechanisms include all AR gene aberrations: amplifications, mutations, splicing variants or changes in co-regulatory genes^19^. Several study showed that AR CNV detection could be useful for predicting treatment response. AR copy number gain is predictive of early resistence to abiraterone and enzalutamide and is associated to worse OS and PFS regardless of prior chemotherapy status^23,24^. Recently, AR-gained patients treated with docetaxel have shown shorter OS and PSF than AR normal but longer response to docetaxel than to enzalutamide or abiraterone^25^. Similarly, AR gain was associated with shorter OS in patients receiving cabazitaxel as third-line treatment^26^.

Our analysis showed an increased, albeit not significant, risk of 31% of detection of AR gain in patients with GG 5 at primary tumor compared to a risk of 20% of those patients with GG 2–4. Then, we evaluated the predictive role of AR copy number detection in patients with GG 2–4 and GG 5 mCRPC who underwent a first-line treatment with docetaxel or abiraterone or enzalutamide.

In the present study, patients with AR normal at baseline showed benefit on median OS and PFS with docetaxel or abiraterone or enzalutamide independently from GG of the primary tumor. In patients treated with abiraterone or enzalutamide, the presence of baseline AR gain and GG 5 correlated to a shorter median OS compared to patients with AR-gained and GG 2–4. Our results indicated no difference in median OS and median PFS in patients treated with docetaxel with AR gain according to GG 5 and GG 2–4. AR normal patients may benefit from docetaxel as well as abiraterone or enzalutamide regardless GG 2–4 or GG 5.

The results of this study confirm the previous evidence that AR-gained patients would obtain greater benefit from docetaxel compared to abiraterone or enzalutamide. Among patients with AR CNV gain, the presence of GG 5 identifies a subgroup of patients with poor prognosis, which could benefit from front-line docetaxel instead of an AR-directed drug.

Recently, Conteduca et al. evaluated the association of plasma AR CNV in combination with 18F-fluorocholine (FCH) uptake on positron emission tomography/computed tomography (PET/CT) and other routinely obtained circulating biomarkers with outcome, in order to perform a better prognostication of mCRPC patients^27^. This study demonstrated that plasma AR CNV, FCH-PET/TC parameters, and some clinical factors (presence of visceral metastasis, neutrophil–lymphocyte ratio and serum chromogranin levels) can be considered as independent predictors of overall survival. In this study, GS did not correlate with clinical outcome to AR-directed drugs. However, patients were stratified according to GS ≥ 8 or < 8 and patients with GS 9–10 were not separately considered and compared to patients with GS ≤ 8^27^.

Our study suggests that AR determination could be a useful biomarker for treatment selection in GG5 mCRPC. Although, we recognize some limitations of our study due to its retrospective nature and the number of patients and events. Our findings have not been validate in a multivariate analysis and in an independent cohort. Therefore, this is an exploratory analysis, which warrants to be validated by larger prospective studies.

## Materials and methods

### Study cohort and design

**Figure 1:**
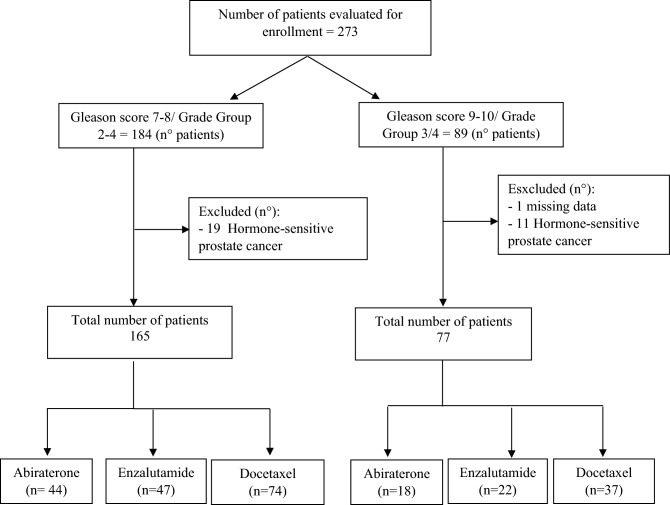
Patients enrolled in the study and type of treatment carried out.

In two single centre prospective observational studies (IRST B048 and IRST B073), we identified 273 men with mCRPC and GS at diagnosis of 7 to 10 (GG 2 to GG5) treated with abiraterone/enzalutamide or docetaxel as first-line treatment from from January 2007 to March 2019. Patients with hormone-sensitive disease (no progressive disease with serum testosterone level < 50 ng/ml) and localized disease were excluded from this analysis (Fig. 1). Ultimately, 242 patients were considered eligible and included in this retrospective analysis. Our Ethical Committee (“IRST Ethical Committee”) approved the IRST B048 and IRST B073 single centre prospective observational studies, all patients gave informed consent. Metastatic CRPC patients performed a first line treatment with docetaxel (standard intravenous dose of 75 mg/mq every 3 weeks), abiraterone acetate (1000 mg/die) plus prednisone (10 mg/die) or enzalutamide (160 mg/die) until progression of disease or unacceptable toxicity. Patients who underwent treatment with AR-directed agents (abiraterone or enzalutamide) have been brought together and were considered as a single group of patients. For each treatment group (abiraterone/enzalutamide or docetaxel), clinical-pathological features and treatment outcome were recorded. Age, ECOG performance status, Gleason score, primary treatment information, extent of disease, baseline serum alkaline phosphatase (ALP), baseline serum lactate dehydrogenase (LDH), baseline serum chromogranin and time from initial diagnosis to initial treatment for mCRPC were collected for each patients. Serum prostate specific antigen (PSA) was recorded at baseline, at 1, 2 and 3 months after starting treatment and at nadir value, if occurred. Progressive disease (PD) was defined according to the Prostate Cancer Clinical Trials Working Group 3 (PCWG3) guidelines as radiographic evidence of new or enlarging lesions by bone scintigraphy and computer tomography (CT) or magnetic resonance (MRI) imagines^28^. Clinical deterioration during treatment was considered a PD criteria as well as radiographic evaluation. GG/GS at initial diagnosis was based on the interpretation of the pathologist where the biopsy was performed and was not centrally reviewed. It was used both GG and GS systems to describe tumor grading.

### Molecular analysis

#### Plasma collection and processing

Baseline peripheral blood samples of each first line treatment from 164 patients were collected in 10 ml plasma EDTA tubes. Peripheral blood samples were collected within 7 days before treatment initiation, blood samples were centrifuged at 1000 × *g* for 15 min to obtain plasma then stored at − 80 °C. Transferring only the upper part of the supernatant reduces the risk of cell or cell debris contamination.

#### DNA isolation and quantification

DNA was extracted using QIAamp Circulating Nucleic Acid Kit (Qiagen, Milan, Italy) according to the manufacturer’s instruction, using 1 ml of plasma. DNA was quantified by a spectrophotometer (Nanodrop ND-1000, Celbio, Milan, Italy) using 2 µl of DNA.

#### Digital PCR analysis

Copy number analyses were performed by QuantStudio3D digital PCR (dPCR) System (Thermo Fisher Scientific) in a duplex assay using FAM and VIC fluorescent probes. AR copy number (AR CN) was evaluated with two assays (*AR1*: Hs04107225; *AR2*:Hs04511283) and two reference genes were selected as control genes: *RNaseP*, TaqMan Copy Number Reference Assay, and *AGO1*(Hs02320401), modified with VIC-labeled probe. DNA samples from three healthy male donors were pooled and used as calibrator.

Data were analyzed using QUANTSTUDIO 3D ANALYSISSUITE CLOUD Software (THERMO FISHER SCIENTIFIC). The average number of copies per reaction microlitres was determined using Poisson distribution. A ratio of target copies and reference copies was measured for each sample, then a ratio between sample and calibrator was calculated. Cutoff value identified was > 2.01 for gain^23–27^.

### Statistical analysis

In this study, categorical and continuous variables were summarized by frequence, median and interquartile range (IQR), respectively. PFS was calculated from the first date of treatment to the date of progression of disease or last tumor evaluation. OS was calculated from the start of therapy to death or last follow up date. PSA reduction ≥ 50% from baseline value during treatment was defined as PSA response rate. Kaplan–Meier method was used to create survival curves, which were compared using the log rank test. We performed univariate Cox regression analysis to correlate Gleason score and other potential biomarkers as predictor of PFS and OS, calculating hazard ratio (HR) and its confidence interval (CI) 95%. Logistic regression analysis was perform to assess odds ratios (OR) and 95% CI of PSA response. All p-value were two sides and all p-values < 0.05 were considered statistically significant. Statistical analyses were executed using SAS 9.4 software (SAS Institute Inc., Cary, NC, USA).

### Ethics approval and consent to participate

The local ethics committee (CEROM) approved the study protocol. The study was performed in accordance with the Declaration of Helsinki.

## Data Availability

The data used to support the fundings of this study are available from the corresponding author upon request.
